# A cross-sectional evaluation of acceptability of an online palliative rehabilitation program for family caregivers of people with amyotrophic lateral sclerosis and cognitive and behavioral impairments

**DOI:** 10.1186/s12913-022-07986-4

**Published:** 2022-05-24

**Authors:** Lene Klem Olesen, Karen la Cour, Heidi With, Annette Faber Mahoney, Charlotte Handberg

**Affiliations:** 1 National Rehabilitation Center for Neuromuscular Diseases, Kongsvang Allé 23, 8000 Aarhus, Denmark Denmark; 2 Department of Public Health, Faculty of Health, Aarhus University, Vennelyst Boulevard 4, 8000 Aarhus, Denmark; 3 Research Unit of User Perspectives and Community-Based Interventions, University of Southern Denmark, W.P Windslovparken 15-19, 5000 Odense, Denmark

**Keywords:** Family caregiver; ALS; cognitive impairments, Behavioral changes, Support, Palliative rehabilitation, Acceptability, Intervention, E-health, Feasibility

## Abstract

**Background:**

Amyotrophic lateral sclerosis (ALS) is a progressive fatal neurodegenerative disease. Around half of the population with ALS develop cognitive and/or behavioral impairment. Behavioral changes in persons with ALS are perceived as the strongest predictor of psychosocial distress among family caregivers. Interventions aiming to support family caregivers are emphasized as important in relation to reducing psychological distress among family caregivers. Successful healthcare interventions depend on the participants’ acceptance of the intervention. Therefore, this study aims to evaluate the acceptability of a new online palliative rehabilitation blended learning program (EMBRACE) for family caregivers of people with ALS and cognitive and/or behavioral impairments.

**Methods:**

A qualitative cross-sectional design using the theoretical framework of acceptability to evaluate acceptance of the intervention based on data collected through individual in-depth interviews and participant observations. Individual interviews were conducted in 10 participants post-intervention and participant observations were recorded during virtual group meetings among 12 participants. A deductive retrospective analysis was used to code both datasets in relation to the seven constructs of the theoretical framework of acceptability: affective attitude, burden, ethicality, intervention coherence, opportunity costs, perceived effectiveness, and self-efficacy. The theory of sense of coherence by Antonovsky informed the development and design of the intervention and interviews. The study adheres to the COREQ (consolidated *criteria* for reporting qualitative research) guidelines.

**Results:**

Within the seven constructs we found that affective attitude addressed the meaning and importance of peer support and focused on the participants’ needs and challenges. Burden referred to technology challenges, time pressure, and frequent interruptions during meetings. Ethicality concerned transparency about personal experiences and the exposure of the affected relative. Intervention coherence referred to a shared destiny among participants when they shared stories. Opportunity costs primary concerned work-related costs. Perceived effectiveness referred to the usefulness and relevance of peer support and the meetings that brought up new ideas on how to approach current and future challenges. Self-efficacy involved the motivation to learn more about ALS and ways to cope that were accommodated by the convenient online format.

**Conclusions:**

The findings showed that the participants favored peer support and the videos that reduced feelings of loneliness and frustration but also confronted them and provided knowledge on future challenges. Further research should explore the benefits of the program and the meaning of online peer support among caregivers of people with ALS and cognitive and/or behavioral impairments.

**Trial registration:**

Retrospectively registered on November 20th, 2020. ID no. NCT04638608.

**Supplementary Information:**

The online version contains supplementary material available at 10.1186/s12913-022-07986-4.

## Background

Amyotrophic lateral sclerosis (ALS) is a devastating progressive neurodegenerative disease that has prominent non-motor manifestations like cognitive and behavioral impairments [1]. The discussion of the ALS and frontotemporal dementia (FTD) continuum has been retold and are now described as two distinct entities [2]. Cognitive and behavioral impairments in ALS are associated with more rapid progression and poorer prognosis and risk of death that is 2 to 2.53 times higher than in unimpaired controls [3]. Cognitive, emotional, and psychological impairments in ALS may cause alterations in certain cognitive functions such as executive functions, verbal fluency, language, and verbal memory [4]. Moreover, impairments and abnormal and inappropriate behavior, like apathy, loss of manners, aggression, and being tactless, are not uncommon in persons with ALS with the cognitive/behavioral variant of FTD [5, 6].

Research shows that behavioral changes are the strongest predictor for psychosocial distress in family caregivers (hereafter caregivers) of people with ALS [7, 8]. Not only do the cognitive and behavioral impairments increase the burden and the anxiety on caregivers, but they also affect their well-being [9–11].

**Fig. 1:**
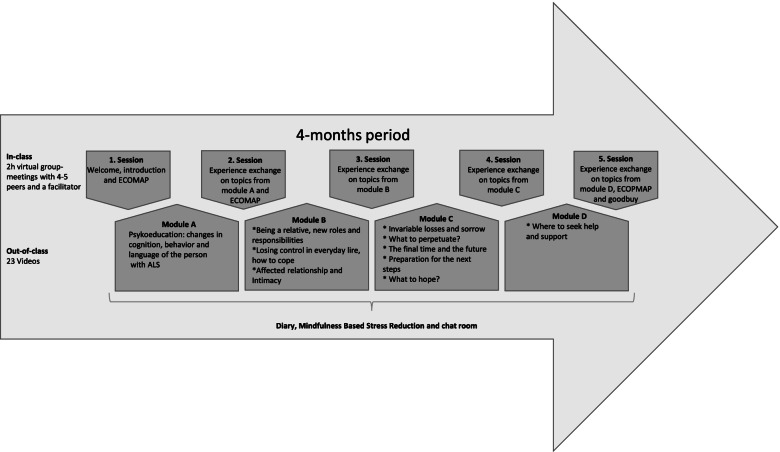
The EMBRACE intervention. A 4-month online palliative rehabilitation blended learning program for family caregivers of people with ALS and cognitive and/or behavioral impairments. The intervention was facilitated by an experienced healthcare professional from the Danish National Rehabilitation Center for Neuromuscular Diseases, who is a registered nurse and certified family therapist with 15 years of experience working with persons with ALS and their families

Caregivers of people with ALS and FTD provide care with a tremendous resilience, compassion, and devotion [12], which is why caregivers need individual time-targeted psychosocial support, containing education and management of challenging symptoms [13]. However, the burdened caregivers frequently refrain from seeking or accepting support due to the difficulty of balancing their personal time with their caregiving responsibilities [14, 15]. There is currently no cure for ALS or the cognitive/behavioral impairments, and two reviews on palliative care in motor neuron diseases (like ALS) therefore advocate for structured support of caregivers in the form of counseling, support groups, and a crisis management system (before and after death of their relative) [16, 17]. Caregivers of people with ALS are likely to experience greater psychological well-being and quality of life from combined psychoeducational support and mindfulness [18]. Similarly, active planning within a multidisciplinary care setting provides an avenue for caregivers of people with ALS and FTD to proactively cope with cognitive/behavioral impairments that will induce improved care and reduce the risk of caregiver burnout [1]. A rehabilitation program for people with ALS and their caregivers has been shown to have a positive effect on the participants’ incentive to understand the disease and benefit from peer support [19]. However, due to the heavy burden and demands caregiving of people with ALS and FTD places on the caregivers [13], it is important to take the caregivers’ time into consideration by using videoconferencing [20]. Research suggests that blended care in the form of combined face-to-face and online healthcare can help bridge the gap between the need for support, information, and lack of time among caregivers of people with ALS [21]. However, some challenges remain because successful implementation of healthcare interventions depends on the recipients’ acceptance of the intervention [22–24]. For recipients to adhere to the intervention and benefit from the improved clinical outcomes [22, 25], it is necessary to develop intervention programs that are accepted by caregivers of people with ALS and cognitive and/or behavioral impairments (PALS/Cis). Hence, we developed the EMBRACE intervention, a 4-month online program aimed at supporting the ability of caregivers of PALS/Cis to handle everyday challenges related to the care of PALS/Cis (Fig. 1). The aim of the present cross-sectional study was to evaluate the acceptance of a new online palliative rehabilitation program (EMBRACE), a blended learning program developed for caregivers of PALS/Cis.

## Methods

### Theoretical framework

The framework on developing and evaluating complex interventions in healthcare from the UK Medical Research Council, the theoretical framework of acceptability (TFA) and the theory of sense of coherence by Antonovsky were used to evaluate the acceptance of EMBRACE [26–29]. According to the updated guidelines from the Medical Research Council, acceptability is important to address during the initial stage of the intervention development [30]. The TFA framework consists of seven constructs: affective attitude, burden, ethicality, intervention coherence, opportunity costs, perceived effectiveness, and self-efficacy that were used to evaluate acceptability in a concurrent and retrospective view [27]. The TFA informed the development of an observation guide, the analysis, and writing up the findings based on the seven constructs [27]. The theory of sense of coherence guided the development and design of the intervention and interviews [29]. The theory focuses on three core components, comprehensibility, manageability, and meaningfulness, that should be present in order to cope and experience life as coherent, thereby reducing stress [29].

### Design and setting

A qualitative cross-sectional design using the theoretical framework of acceptability to evaluate acceptance of the intervention based on data collected through individual in-depth interviews and participant observations [27].

The study was carried out online through the platform Simplero, and group meetings were run with Microsoft Teams. The study was embedded at the National Rehabilitation Center for Neuromuscular Diseases (RCFM) in Denmark [31]. RCFM is a national, highly specialized private outpatient hospital financed by the government, with rehabilitation services free of charge for its patients [31, 32]. RCFM offers highly specialized advice and counseling to persons with neuromuscular diseases, their families, health professionals, professional caregivers, and rehabilitation specialists [31]. Public neurological hospital departments refer about 95–97% of people with ALS to RCFM [31]. The professionals at RCFM are organized in multidisciplinary teams consisting of occupational and physiotherapists, nurses, doctors, psychologists, and social workers [31]. To provide rehabilitation on the patients’ terms and to get as much insight into the patients’ everyday lives as possible, most of palliative rehabilitation by the professionals at RCFM is performed in the homes of the persons with ALS [31].

### Intervention

EMBRACE had a blended learning format, combining both videos and virtual group meetings. The content rests on evidence- and experience-based topics identified in a qualitative study on challenges and needs among caregivers of deceased PALS/Cis [33]. We developed and recorded 23 videos based on topics associated with caregivers’ challenges and needs. The participants received a diary before starting the invention and were encouraged to take notes and write down their thoughts during the intervention. The participants were asked to make ecomaps three times during intervention as a means to explore potential supportive relations that could be beneficial during the disease-trajectory and after the death of the PALS/Cis. They were also offered customized Mindfulness Based Stress Reduction videos. The diary, ecomaps, and the mindfulness videos were not used as data. In addition to the empirical evidence and experience base, the theoretical lens of sense of coherence strengthened and targeted the content in EMBRACE to meet the caregivers’ need for comprehensibility, manageability, and meaningfulness [29]. EMBRACE consisted of three groups, each of which included 4–5 participants, facilitated by the third author. EMBRACE was developed and carried out by the first and third authors, who had extensive knowledge of the field under research due to working as healthcare professionals at RCFM. This team received regular professional group supervision during the delivery of the intervention.

### Characteristics of participants and sampling

Participants were sampled based on the following inclusion criteria: [1] caregivers (partners and spouses) living with a person diagnosed with ALS referred to RCFM who had received an initial visit from healthcare professionals from RCFM, [2] caregivers who were able to speak and understand Danish, and [3] caregivers of persons with ALS with a cut-off score ≥ 22 on the Amyotrophic Lateral Sclerosis-Frontotemporal Dementia-Questionnaire (ALS-FTD-Q), a validated questionnaire containing 25 items, the total score ranging from 0 to 100, with higher scores indicating more behavioral changes [34]. A cut-off score ≥ 22 on the ALS-FTD-Q indicated mild behavioral change of the person with ALS [34]. Caregivers were encouraged to invite a relative to accompany them throughout the intervention. The companion could not be an affected relative. Two caregivers chose to invite an adult relative to accompany them.

**Fig. 2:**
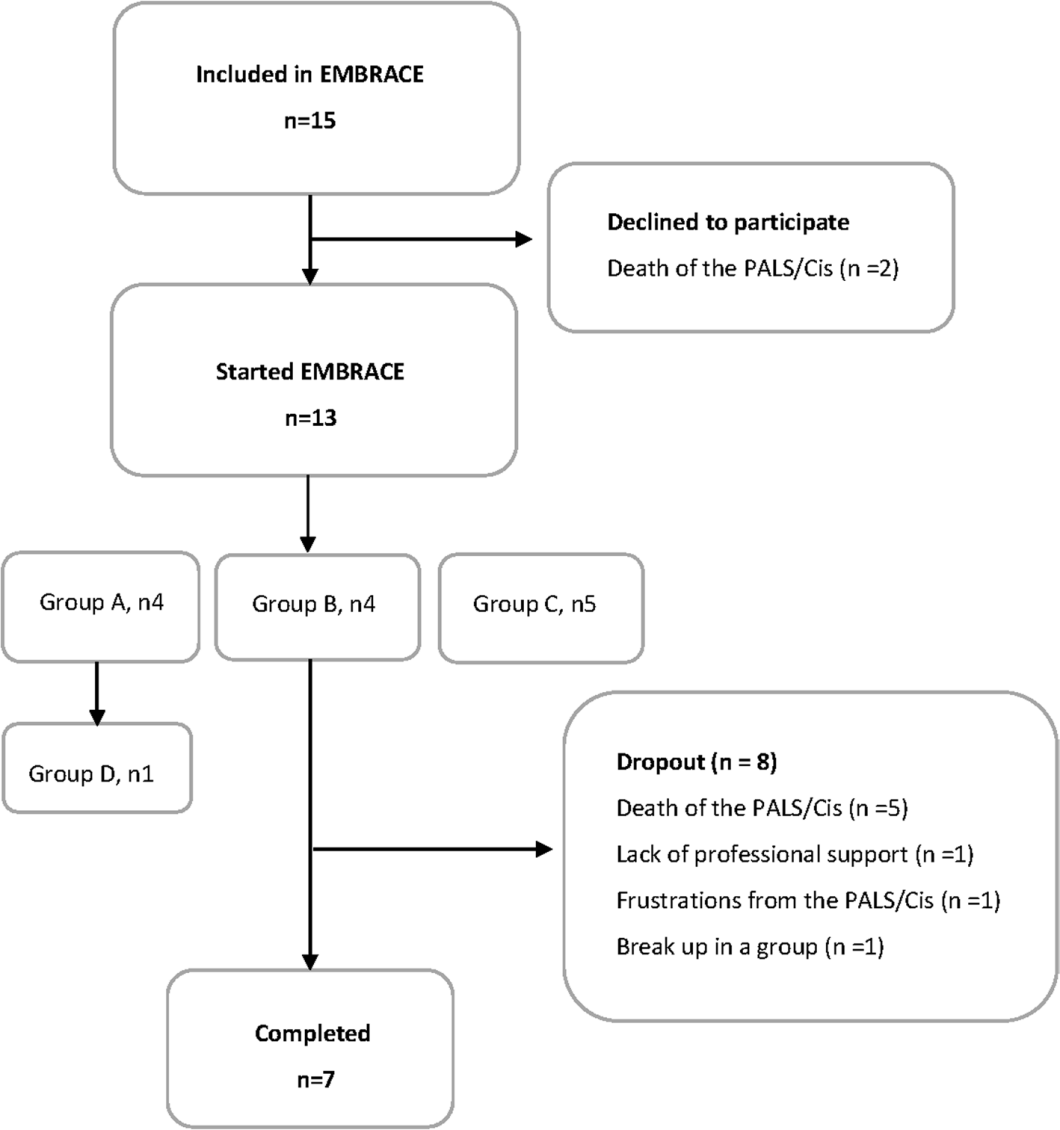
Participant flow diagram. Overview of allocation and numbers of participants in each group, including numbers of and reasons for participants dropping out

**Fig. 3:**
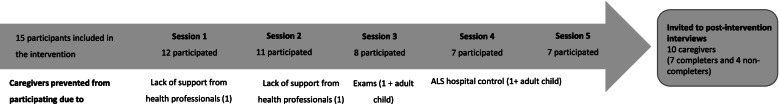
Overview of participants in group session, participants invited to interviews post-intervention, and reasons for non-participation

A two-step sampling process was performed for the intervention. First, healthcare professionals from RCFM helped identify persons with ALS referred to RCFM up to September 8, 2020, who met the first and second inclusion criteria. Next, invitations containing information about the intervention program and the research project were sent to persons with ALS and caregivers, 208 in total. Thirty-one caregivers contacted the first or third author, wishing to participate. The interested caregivers participated in screenings by phone where they scored their affected relative using the ALS-FTD-Q [34]. A total of 15 participants were included in the intervention (Fig. 2). Participant observations during the interventions in 16 virtual group meetings were obtained from 12 of the 13 participants who started the intervention (11 partners and 1 adult child of a parent with ALS) (Table 1). All 15 included participants were invited to participate in interviews about their expectations for EMBRACE prior to the intervention. Eleven of the 12 participants (including non-completers) were invited to participate in post-interviews (Fig. 3). The person who was not invited had just lost a relative who had died of ALS. For this study, we draw on the post-interviews and participant observations. The inclusion for post-interviews was ongoing from September 14, 2020, to February 25, 2021. Ten caregivers out of 11 participated in post-interviews. One did not respond to the invitation.

**Table 1:** Demographic data on the participants based on the dataset from the participant observations and interviews

Participants		(***n*** = 12)
**Gender**	Male	3
	Female	9
**Age**	18-25	1
	39-50	3
	51-55	4
	56-67	4
**Relation**	Married/partner	11
	Adult child of a PALS/Cis^1^	1
**Occupational status**	Working	7
	Early retirement/retired	4
	Studying	1
**Years of ALS-trajectory**	0-2	4
	2-4	2
	4-8	4
	8-12	1
	12-14	1
**ALS-FTD-Q score**	22-30	2
	31-35	5
	36-40	1
	41-46	3
	47-55	1
**Urban**	≥ 80.000	1
**Rural**	≤ 80.000	11

^1^Person with amyotrophic lateral sclerosis and cognitive and/or behavioral impairments

### Data generated

Data were generated by using data triangulation with individual in-depth interviews post-intervention and participant observations during the group meetings. We chose to include participant observations in this dataset to obtain an objective point of view on the intervention [35]. Retrospective participant observations in 16 recorded virtual group meetings were carried out individually by the first, third, fourth (an external health anthropologist), and fifth authors. Each of the 16 meetings lasted for around 2 h and were run with Microsoft Teams. Participant observations were carried out according to a predefined participant observation guide (Table 2).

**Table 2:** Participant observation guide for caregivers of PALS/Cis

The TFA constructs	Elaborative participant observation questions
**Affective attitude**	How do the participants show and express their feelings about the intervention?
**Burden**	How do the participants show and express their perceived amount of effort required to participate?
**Ethicality**	How do the participants show and express the intervention’s fit with their individuals value system?
**Intervention cohesion**	How do the participants show and express their understanding of the intervention and how it works?
**Opportunity costs**	How do the participants show and express their opportunity costs, like benefits, values, or profits that must be given up to engage in the intervention?
**Perceived effectiveness**	How do the participants show and express their experience of perceived effectiveness/or the opposite with the intervention?
**Self-efficacy**	How do the participants show and express their confidence that they can perform the behavior(s) required to participate in the intervention?

This evaluation focused on the execution process and retrospective experiences of acceptability to accentuate the participants’ perceptions and experience of EMBRACE. Therefore, the pre-intervention interviews will be reported elsewhere. The post-interviews are reviewed in the present study. Interviews were carried out by the first author with the seven participants completing the intervention and three non-completers. Interviews with non-completers were carried out to learn about their reasons for withdrawing and potential barriers regarding acceptability. Nine interviews were generated online using Microsoft Teams, and one interview was conducted in-person at the caregiver’s workplace. Interviews were carried out by the first author and were digitally recorded. Interviews lasted between 58 min and 1 h 41 min.

### Observation guide

A participant observation guide composed of seven constructs from the TFA [27] was used (Table 2).

### Interview guide

A semi-structured interview guide composed of open-ended questions was used. The questions focused on the participants’ experiences, attitudes, feelings, preferences, and boundaries regarding the intervention and recommendations for improvements [See Additional file 1].

### Data analysis

**Fig. 4:**
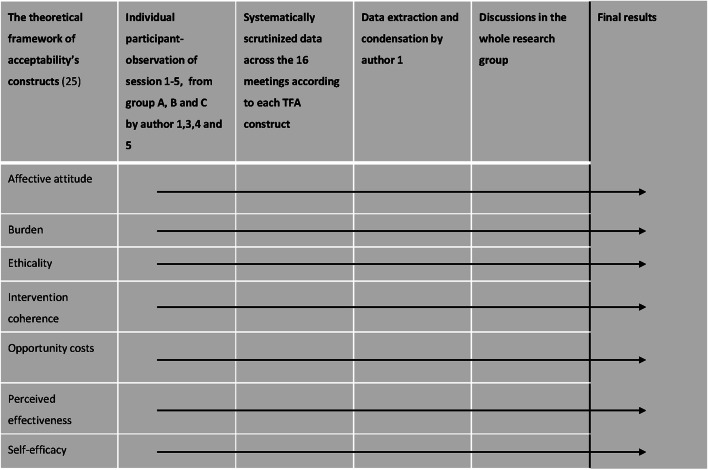
Analysis process of participant observations. The seven constructs of the theoretical framework of acceptability were used: affective attitude, burden, ethicality, intervention coherence, opportunity costs, perceived-effectiveness, self-efficacy [27]

All data were organized and analyzed retrospectively and deductively according to the seven constructs of the TFA [27]. Participant observations were carried out individually by the first, third, fourth, and fifth authors. Each person watched all 16 recorded videos from the group meetings and filled out the predefined participant observation guide for each video. Next, the whole group systematically went through each construct for each video, allowing each person to present their organization of data within the construct. The group then discussed what had been said and whether it was the correct organization according to each construct. Data extraction and condensation related to each of the seven constructs across the 16 meetings was subsequently undertaken by the first author, and the extract was discussed with the entire group (Fig. 4).

Interviews were transcribed verbatim, then read and individually deductively coded according to the seven constructs in NVIVO^12^ by the first and fifth authors [27]. The selected codes and phases were then discussed in the whole research group in terms of which codes were most fitting according to the TFA constructs [27].

## Results

Feasibility results on acceptance of the EMBRACE intervention are structured by the TFA constructs and presented below [27].

### Affective attitude

Affective attitude concerned the participants’ feelings toward EMBRACE mainly centering around the group meetings and the impact of the attitude of the other group members. Observation showed that the participants expressed positive thoughts toward the EMBRACE intervention and were grateful for having been given the opportunity to participate. In general, they were positive about the intervention and described the development of relationships with group peers throughout the meetings and the importance of peer support as meaningful.

“I've liked the closed forum where everything has been allowed. It's been pretty liberating to be able to talk about what you are struggling with.” (ID 2G)

“Yes, I also found support in listening to each other’s stories, and I thought ‘Ah, I’m not the only one who feels like that. In a way, it’s a good thing. Not that you want it for other people, but it’s nice to know that you’re not alone.” (ID 2C).

Participant observations showed that the participants talked about looking forward to the meetings, which they said were a welcoming break from their everyday lives, which were otherwise filled with various activities, work, care, and support for the PALS/Cis. Generally, the participants found that the meetings were characterized by a special atmosphere and that there was a mutual understanding and sympathy for each participant’s everyday challenges. The meetings were perceived as a common ground where the participants felt free to ask questions regarding things they were worried about. For instance, the less experienced participants embraced the lived experiences of the more experienced participants by expressing the importance of and their appreciation for learning from peers, which they found useful as preparation for the difficult time ahead of them. The more experienced participants showed consideration for the feelings of the less experienced participants, who they knew would soon learn the harsh realities of living with a spouse in the advanced stage of the disease, which they expressed by showing their sympathy for and deep understanding of the everyday challenges these participants faced. Directing their focus from the well-being of the PALS/Cis to their own needs was also seen as a major benefit of the intervention because everything around them usually concerned the PALS/Cis.

“When I watched some of the videos, I thought ‘oh my God, it’s me in that video.’ It’s me talking. And it’s been like, I know it’s strange to use the word ‘nice’, because there is nothing nice about it, but, well it gives you peace of mind. You know, relief and peace because like ‘well, there’s actually something I’ve got under control’.” (ID 2E).

Although, several participants described feelings such as tension or having stomach cramps before and during the first meeting, these feelings were later replaced by feelings of relief, peace, thankfulness, and being less lonely and less frustrated.

“I feel relieved when I leave the meeting. It’s something about the way I breathe. There is room to breathe.” (ID 2B).

One participant did, however, find the meetings exhausting and causing stomachache, which made it difficult for her to listen and open up to the other participants. On the other hand, she said she was comfortable with just listening to the other participants because she recognized what they were saying.

### Burden

Burden contained the perceived amount of effort that was required to participate. In general, participant observations revealed that the challenges the participants faced during the intervention were related to technology issues, interruptions during meetings, lack of time, and difficulties reading body language during the online meetings. Technology problems included unstable internet connections causing the screen to freeze, missing images of anyone but the person speaking, echoes, and overheated devices. Although the participants had secluded themselves from their surroundings during meetings – in bedrooms, private offices, the workplace, a car, or children’s or parents’ houses – they were sometimes interrupted by phones ringing, children entering the room, requests to assist the PALS/Cis, finding a charger for the computer, and having to change location in the home.

“And then again, with a poor internet connection, and then one thing happens after another. Well, and then there’s just the thing about having my husband in the house, right? Well, you can’t just … I’ve had to close three doors and turn on the TV in the living room downstairs to make sure that he can’t hear me.” (ID 2K).

The interruptions shifted the attention of the participants from the meetings to the situation in the home. Because of time pressure, the participants had difficulties giving priority to themselves and talked about finding it hard to settle down to participate in the meetings and watch the videos between the meetings.

“Well, at that point I thought that maybe an hour would be enough, because I actually felt exhausted. You had to be ready for it, and you had to compose yourself and find the time, and it had to fit into your daily schedule.” (ID 2F).

The meetings were described as intense, which fatigued some participants, but on the other hand, they did not want to reduce the length of the meetings.

“It’s difficult to deal with such emotional themes for 45 min, and get everyone to say something. On the other hand, it’s also extremely difficult to set two hours aside when you are at home with a sick spouse and a care team. Your presence is frequently required, so you must go back and forth during the meeting. You’re interrupted. And then something else happens, and you have return to the subject being discussed, but can you do that mentally? It probably can’t be done any other way when you’re in this situation.” (ID 2I).

Mutual apprehension between the participants was also referred to as an important factor.

“I think it was because we hadn’t picked each other. Because we all know that if you, like, know the others, then you know who you get along with. So, it was like being together with people who were forced on you, because you hadn’t chosen them.” (ID 2F).

Despite finding the virtual platform convenient and easy to operate, some participants said they would have preferred physical meetings because the virtual format made it difficult to read body language and have casual conversations.

### Ethicality

Ethicality concerned the extent to which EMBRACE had a good fit with the participants’ value systems. The meetings became an intimate room for asking other participants direct and confronting questions as well as a room for telling their personal experiences about everyday challenges regarding the PALS/Cis, such as how to do deal with apathy from PALS/Cis or deal with great frustrations due to living with PALS/Cis who had no recognition of their disease.

“It’s really like you’re asking people; ‘Why has your husband chosen to live?’ It’s really a difficult and big question. That’s what I’ve been struggling with for a long time.” (ID 2K).

For several participants telling the truth involved many ethical issues which they had not discussed with anyone else.

“You expose your spouse in a completely different way, right? And the thoughts you share are not something other members of the family should hear.” (ID 2K).

For instance, this could be a wish for a quick disease trajectory because of the degrading situation for the PALS/Cis, but at the same time not wanting to lose a spouse. However, this was difficult for one participant to relate to.

” It’s been hard. Because members of my group were quite negative, and it drained my energy. I think they had a hard time finding something positive to say. And I couldn’t relate to how they somehow wanted it to come to an end. It was a completely different world for me (giggles). Yes. I felt they almost blamed their spouse for falling ill. Presumably, leaving them as the strong ones. For me it was unfamiliar land, I didn’t understand them.” (ID 2F).

Several participants described having no one else to share such thoughts with, as they did not expect people without personal experience with a PALS/Cis to understand their situation and feared they would be judged as unsympathetic.

“Well, I think that the honesty – that honesty – that you don’t have to beat around the bush because you’re scared, you know... That it’s actually okay to say ‘right now it really sucks,’ you know, ‘because so and so and so’. People know what it means, it’s not just because I use bad language, it’s because I’m being honest. You don’t have to be afraid to tread on someone’s toes or eh … People understand you and they accept it, right. But as I said before, I wish the intervention would have been longer.” (ID 2O).

Some participants felt that sharing personal stories from everyday life in the group meetings would expose the PALS/Cis in a negative but nevertheless truthful way. During meetings the participants shared details about private dilemmas and challenging situations even though it meant exposing themselves and their partners. When asked to think of a dream scenario of how everyday life could be, some participants found it difficult because they had trouble putting themselves first and said that they were not the one that was soon to die.

### Intervention coherence

Intervention coherence concerned the extent to which the participants understood EMBRACE and how it worked. The participants expressed an understanding of the purpose of EMBRACE, by underlining the meaning of the intervention targeting their needs as relatives, but participant observations showed that sometimes they had to be reminded to focus on their own needs and challenges and not on those of the PALS/Cis.

“I think EMBRACE is really good because it offers information. It prepares you for everything that’s going to happen. I think that’s important. You become prepared for what you’re probably going to face. Well, so you’re prepared to act.” (ID 2I).

“Getting a forum as a relative and gaining this knowledge. Because I wouldn’t have gotten any knowledge if I hadn’t searched for it myself. So, what turns up as a structured offer in such a course is really good, because the disease IS serious!” (ID 2I).

The participants exemplified how the intervention had worked, for instance, by pointing to the supportive element of peer support and insight into various experiences on how to handle or prepare for possible future challenges.

“And group meetings, that’s the thing when you hear from other people, that they are … I’m reassured that I’m not alone in the world, that there are others whose lives are as hard as mine. I also get, I also discover that there are others that are just getting started.” (ID 2A).

“No, but just talking to someone who knows how it is, and how it can be, and how much the disease takes up your life and how you sometimes feel like throwing up and think ‘I don’t want to do this anymore, can it please just end’. Sometimes you just feel like that. Of course, people don’t understand you when they’re not in the middle of it, so you don’t say it aloud. But it’s actually okay to speak out to someone who’s in the same situation, because we’ve all felt like that now and then.” (ID 2C).

The participants thereby gained a better understanding of their own situation and challenges and how to handle these. Participants emphasized the common thread between relevant topics in the videos and the group discussions that prompted emotional conversations that they could not have had with family and friends. Despite being different in terms of personalities, values, challenges, and stages of their partner’s disease, the participants’ common situation of living with a PALS/Cis made it possible for them to better understand, relate to, and support one another.

### Opportunity costs

Opportunity costs were related to the extent to which benefits, profits, or values must be given up to engage in EMBRACE. This construct was not one of the main focus areas, but two conditions were brought up. These concerned having to take time off from work and cancelling a study group meeting to participate. The participants generally gave high priority to the meetings although their busy lives made it hard for them to find the time.

“It suited me fine. Because of the COVID-19 pandemic I was working from home, so I could fit the meetings into my schedule and work flexible hours.” (ID 2I).

“When it’s busy at work, the driver [a colleague] walks around singing. But that’s the way it is. That’s the only way for me to participate. I couldn’t participate from home. That’s not possible. Well, that’s nonsense, because I could have said to myself; ‘I’ll go to another room and close the door and the care team can yell and scream as crazily as they want.” (ID 2A).

### Perceived effectiveness

Perceived effectiveness concerned the extent to which EMBRACE was perceived as likely to achieve its purpose. The participants found the intervention useful and relevant, especially stressing the importance and benefits of peer support and targeted videos.

“I’ve learned something every time. I really have. Also, my understanding of the disease and all the issues it raises. Well, in a way, I wouldn’t say, I’ve calmed down inside, but I think I’m more prepared for what’s going to happen. Emotionally, too. Because you have seen other group members who are at a more advanced stage of the disease and how they have handled it. However, we are all different and deal with such situations in different ways. You must remember that. But it has certainly helped me, because I have begun to search for who I am and to be better prepared emotionally as things happen.” (ID 2H).

“I think that the thing about us being at different stages of the disease, I think that’s really good. I don’t think there would be anything to learn from it if all our relatives had just been diagnosed, because what would we talk about? I think that (being at different stages) is really good, and I think that those of us that are new learn a lot from hearing the stories. A great deal actually.” (ID 2K).

They found the topics, format, and discussions so useful that the did not want the intervention to end. They said that they felt included in a community of shared destinies where sympathy for each other’s everyday life challenges was emphasized.

“Well, to be seen, heard, and understood. I think that means a lot. I mean what I learn from it. You know, you can – I have a huge network – and you can talk to them, but it’s in a different way, and they have another frame of reference than the one you have, as a relative. So, meeting others means a lot to me.” (ID 2B).

In spite of difficult and sorrowful conversations and an initial lack of energy, participants said that they felt the meetings were invigorating; removed some of their burden, frustrations, and loneliness; and provided them with new ideas on how to approach current or future challenges. Additionally, the videos gave rise to reflections and understanding of targeted topics, thereby intensifying the focus on the participants’ needs and challenges.

“I think it was good, and that it (EMBRACE) covered many different things – both practical and emotional things – and well, all the different challenges that you have faced or will face.” (ID 2H).

For some, writing notes and reflections in their diary was a way to reduce stress by helping them to “get things out of their mind” and not constantly having to remember everything. From participant observations, we noted that several participants talked about experiencing bodily relief, feeling calmer, more peaceful, and being able to breathe easier.

### Self-efficacy

Self-efficacy concerned the participants’ confidence that they could perform the behavior required to participate. They described different behaviors and how these either enhanced or hindered their participation in EMBRACE, like having difficulties in asking confronting questions or figuring out how to express oneself.

“I haven’t done anything wrong, right? I’m really bad at that. I mean I’m really bad at blaming myself for everything. But I’ve also become better at realizing and accepting it, and I’m working on doing something about it. It’s a huge process, and I’m not sure that I’ll ever cross the finishing line; I know that, but it’s a relief to know that it’s there.” (ID 2E).

The virtual format made it possible and easy to attend the meetings and watch the videos, which enhanced participation. The flexible and non-demanding nature of the meetings helped the participants attend without having a guilty conscience about not being “prepared”, not having watched the videos beforehand, etc. The diary made it easy to take notes for those who found this valuable. Motivation for wanting to learn more about how to handle challenges related to living with a PALS/Cis as well as contributing to research to support future caregivers also enhanced the participants’ engagement in the intervention.

“It was great having the opportunity to talk, but I also found it difficult. Although I spoke very bluntly in that context, it was hard. It was hard for me to assess what was the right thing to say. It was very difficult because I wanted to give something to the others, but did I do that? Or was it a scare story, or what was it, right?” (ID 2I).

Lack of concentration and poor memory were mentioned as hindering factors for their ability to focus and remember things said in the videos.

“Then I will try to download them, because I think that the one with the preacher – there were so many, many, many things that you – well that were hard to take in all at once. And that’s exactly what each video is – how do I put this – it’s unique, right, but’s also consuming. First you must watch it, then work through it and then again convert it into something you can use. So, it’s not done in just one afternoon, is it?” (ID 2O).

Their desire to help the members of their group caused them to share their personal stories in order to prepare these members for the future. However, participant observations revealed that this sometimes involved talking about the affected relative instead of their own personal challenges. Some participants praised others for their eloquent way of describing their problems while not holding back their own thoughts. Moreover, they became more courageous during the series of meetings, asking each other more personal questions and discussing serious issues.

“Well, I could listen and then I could ask. When we’d meet once or twice it was okay to ask those questions – about practical matters but also about difficult things. And one thing I could also really use it for was that I could use it to, like, think about ‘how am I as a person in this (situation)’ compared to ‘how are the others’.” (ID 2H).

Some participants talked about gaining new personal insights during the meetings and how the meetings changed their ways of understanding and dealing with different situations.

## Discussion

This study sheds light on the acceptance of the EMBRACE intervention from the perspectives of caregivers of PALS/Cis. We found factors related to all constructs of the TFA, but some were more prominent than others. The discussion is structured according to the TFA constructs.

Regarding affective attitude, the participants generally reported very positive experiences about participating in EMBRACE and for the opportunity to engage with peers. Our study showed that the participants made use of their peer’s different perspectives regarding caring for a PALS/Cis to prepare for future challenges. Similar findings were observed in a recent study on a psychoeducational intervention for persons with ALS and their caregivers [36]. They found that peer-support was one of the two main reasons for utility of the intervention [36]. In other studies, peer-support has been shown to lead to camaraderie, comparisons, and hope [37]. Comparisons with people who are dealing with things that are experienced as worse or more difficult than what ALS patients dealing with has further been demonstrated to be helpful for ALS-patients to feel better about themselves and their situation [37]. Trying to balance between handling everyday challenges and not knowing what will come next seemed to use up a lot of the participants’ resources. This is in line with prior studies showing that caregivers face the conflict of trying to be prepared for the future while being overwhelmed by the issues of caring throughout the progression of the illness and coping with uncertainty [38, 39]. However, the participants in the present study embraced the stories from the more experienced participants despite this opening a potential black box regarding the later stages of the disease. Locock and Brown [37] found that some caregivers and ALS-patients chose isolation as a deliberate defense strategy to protect themselves from facing a potential future situation while others valued social interaction with peers. For our participants sharing thoughts on hopes and sorrows with peers during group meetings broke down some of the barriers and fears concerning the future. A report on caregivers’ preparation for the death of their relative found that caregivers were plagued with a guilty conscience when thinking about the future [38]. They found that caregivers might be cognitive and behaviorally prepared for the future but not emotionally, due to the situation of living with both hope and fear [38]. The participants in our study looked forward to the meetings and appreciated the focus on their needs and challenges, despite sometimes finding it difficult not to talk about the PALS/Cis. This might be because caregivers tend to regard their own needs as secondary compared to the needs of the PALS/Cis [15].

Burden concerned the technology issues, caregiving responsibilities, and lack of time that affected the participants’ attention toward the elements of the intervention. We found that the blended virtual format created an accessible opportunity for the caregivers to participate despite lack of time, intense meetings, and problems with the technology. Our findings on the benefits of using an online blended learning format showed that it enhanced accessibility and could perhaps bridge the gap between the needs of the caregivers and their lack of time due to caregiving responsibilities and practical tasks. In line with our findings, another study showed that accessibility of the support given was crucial for the increase in self-efficacy among caregivers of ALS-patients [21]. Our findings demonstrate that the participants prioritized attending the meetings because they felt related in a special way to the other group members, who understood their situation and meet their needs for support. Mazanderani et al. (2012) also found that similarities in diagnosis was an important reason for valuing other’s experiences as knowledge [40]. The use of social media has also been shown to increase the connection among caregivers of people with ALS, as well as their attendance and socialization [41]. The sense of distance that can occur between people when communicating through social media can furthermore for some people enable particular forms of computer-mediated distal empathy and still enable interactions and sharing of experiences with peers [40]. However, timely provision of problem-solving coping strategies is important to take into account when mitigating caregiver burden in PALS/Cis [1].

Ethicality was identified as dealing with feelings of guilt regarding the sharing of private challenges and exposing the PALS/Cis, but at the same time not wishing to be judged by peers. The participants placed themselves in vulnerable positions by being transparent about their everyday challenges. However, talking with peers about challenges and future concerns seemed to reduce feelings of guilt, which is consistent with a previous study on caregivers of people with ALS [15]. The authors found that caregivers experienced cohesion when sharing personal experiences and tips with peers who understood their situation and what they were going through, which nobody else in their social network could [15]. Contrary to that study, participants in our study shared intimate challenges with peers, and did not feel that topics like these were too private to discuss [15].

Intervention coherence concerned to what degree the participants found the topics relevant, useful, and empowering in relation to understanding and dealing with their personal challenges. To offer the participants knowledge on the disease, existential factors, resilience, and potential future challenges empowered them to change or moderate their interactions with the affected relative, which was also found in a previous report [18]. Effective caregiving requires that the caregivers receive emotional and practical support which allow them to better manage the different needs of their sick relatives, thereby reducing the overall burden and increasing empowerment [18]. Our study showed that the participants engaged with peers and supported each other in a way that family and friends were not able to do. Despite exposing a vulnerable side of oneself and risking potential tough comments from peers, the participants found the courage to speak up in order to receive advice and support. Reports confirm the benefits of peer support as encouraging mutuality and overcoming feelings of social isolation [42, 43]. We found that the participants were willing to open up and share concerns, which contradicts what De Witt et al. (2019) found in caregivers of people with ALS, where the majority of participants indicated that they would be passive partakers in group sessions and would only read the information and not share personal stories [15]. Studies show that being in the same situation as ALS-patients or caregivers was experienced as beneficial in relation to comparison of progression and challenges, but also confronting in terms of facing reality [19, 37]. A study illustrated that involvement in groups of carers or ALS-patients could change over time as they struggled with their changing needs and fears [37].

Opportunity costs were related to how the participants had to give up work or study groups to participate and were not something that they paid a lot of attention to. This might be influenced by the setting in which the research was conducted, because in Denmark, health and social care is free of charge, and the participants therefore did not experience financial costs in relation to participation. In contrast, studies on caregivers of people with ALS have found that caregivers perceive the uncertainty about their financial futures as stressful, because care responsibilities often compete with work and/or other family commitments [39, 44]. The majority of published studies stem from developed countries, and many studies do not take socioeconomic variables into account, like individuals wealth or national healthcare systems, which makes it difficult to extrapolate results to all countries [45].

Perceived effectiveness concerned the participants’ feeling that group meetings and peer support were invigorating, encouraged mutuality, and removed some of their burden, frustrations, and loneliness, providing them with ideas on how to approach and deal with challenges. The intervention thereby seemed to fulfill its purpose. However, a report by Weisser et al. (2015) shows that caregivers of people with ALS express a need to be encouraged to seek support, timely information, and education, based on personalized care, in order to foster resilience [46]. Nevertheless, we found that targeting information on cognitive and behavioral impairments not only offered an intimate and reflexive environment but was also useful to emphasize the shared destinies and to learn from peers. Caga et al. (2021) also found it particularly important to offer information on ALS and cognitive impairments and problem-solving strategies as part of supporting caregivers of PALS/Cis [1]. Our results showed that some participants found it beneficial to keep a diary during the intervention. Offering caregivers of critical ill persons a diary is important as a means to gain understanding and to cope, and it may also reduce post-traumatic stress disorder, anxiety, and depression among caregivers [47].

Self-efficacy involved how the participants found it convenient and easy for them to participate. The blended learning format and the non-demanding participation seemed to be imperative to accommodate the heavily burdened participants who found it difficult to leave their relative at home alone. These findings are consistent with a previous report highlighting online services, like telehealth, as a way to support caregivers of people with ALS [48]. Telehealth in ALS is often well-received by caregivers, but finances and legislation may hinder telehealth implementation in ALS care [49]. Our results showed that the participants were motivated to learn more about the disease and how to deal with it, but that they found it difficult to assimilate knowledge due to stress, fatigue, and poor memory. However, a recent report showed that knowing too much about the disease trajectory could have a negative effect on caregivers’ experience of burden [44]. Nevertheless, our study adds to the importance and meaning of gaining insight and knowledge from peers to understand and manage the diseases as a caregiver [19]. Our study also adds to the success of complimenting group-based peer support with psycho-educational interventions [50].

Overall, the participants’ acceptance of EMBRACE was related to the opportunity to meet and share experiences with peers, which is in line with the TFA’s assumption that acceptability may impact the behavioral engagement in the intervention [27].

### Strengths and limitations

This study has several strengths. The TFA framework and the theory of Antonovsky proved useful for guiding the interviews, the intervention, and the analysis of data as the TFA offered pre-defined constructs to address a complex phenomenon as acceptability. This helped us design the intervention to increase the sense of coherence and reduce the stress of caregivers of PALS/Cis. The data triangulation of interview and observation data provided us with a rich and nuanced perspective of the participants’ level of acceptance and thereby strengthened insights gained [35]. The participants were interviewed within two weeks after the intervention, which meant their experiences were still on top of their minds. It might have strengthened our findings if the participants were interviewed about their acceptance of the intervention during one of the group meetings, as it would have provided the participants with the opportunity to discuss, share, and elaborate on their perceptions of acceptance of EMBRACE. A potential limitation could be that we did not include the pre-interviews in the present study, which could have given insight into the participants’ expectations regarding the EMBRACE intervention before enrollment. However, we aimed to evaluate the participants’ acceptability of EMBRACE, not their perception of the intervention. Additionally, questions in the interview guide for the post-interviews were not fully analyzed in this study due to the deductive TFA analysis, which was used as an alternative to thematic analysis and could therefore have comprised the empirical data [51]. In the same way that the guide lacked specific TFA questions which might have revealed further perspectives on ethicality and opportunity costs, some constructs were only represented briefly and therefore perhaps not fully portrayed in the current study. However, by using the TFA in the analysis, we were able to access both enhancing as well as restraining issues regarding evaluation of the acceptance of EMBRACE [27].

As to representative credibility, the relatively small sampling of 12 participants reflects firsthand perspectives of 10 participants interviewed on acceptance of EMBRACE but with an overrepresentation of women’s perspectives (Table 1). However, the ratio of men to women with ALS is reported to be between 1 and 2 [52]. Most participants in the present study were between 51 and 67 years of age, and thereby represented the general family caregiver [52]. They represented the full trajectory of ALS, with experiences ranging from months to 14 years. Despite this sampling, there is always more to study, and according to Thorne, there are no such notion as data saturation [35].

According to analytical logic and interpretive authority [35], the first author generated all the data while also being an “insider” with experience of working within the research field. The fact that the first author played a central role before and after the intervention might have entailed the risk of the participants not speaking freely and honestly. The first author did, however, not facilitate the group meetings and therefore were not in direct contact with the participants during the intervention. Furthermore, none of the participants knew her beforehand and during the interviews, they did not hesitate to express pros and cons of their perception of EMBRACE. Finally, the “insider” position made preunderstandings unavoidable which could have increased the risk of missing aspects or misinterpretations in relation to what an “outsider” would find [35]. However, to avoid these risks, the research team was a combination of researchers conducting the intervention and researchers who did not contribute to carrying out the intervention. Moreover, to reduce the risk of “blind spots”, we included an external health anthropologist in the research group, who performed the initial observations. In collaboration the whole team coded the participant observations along with scrutinizing data according to the TFA constructs. Interviews were coded by the first and fifth author and then discussed in the whole research team.

## Conclusion

This study evaluates the acceptance of the online palliative rehabilitation blended learning program, EMBRACE, from the perspectives of caregivers of PALS/Cis. Results indicate that the intervention supported caregivers of PALS/Cis in dealing with everyday challenges in relation to a PALS/Cis and reduced their experience of guilty conscience, fear, loneliness, uncertainties, and gave insights into ways of dealing with everyday challenges now and in the future that they could not have gained elsewhere. A special atmosphere in the group meetings fostered greater social connectedness and feelings of belonging to a group among the participants, thereby reducing feelings of loneliness. The results demonstrate facilitators as well as barriers to consider when offering targeted online group-based interventions for caregivers of PALS/Cis. Attention toward the participants’ experience of affective attitude, burden, ethicality, opportunity costs, and self-efficacy should be especially considered when targeting caregiver support in order to develop an acceptable and useful supportive intervention. The use of the TFA helped identify issues within the seven constructs of acceptability that were useful for informing modifications in the design of EMBRACE. Future research should investigate the perceived impact from participating in EMBRACE and the effect of online peer-support for caregivers of PALS/Cis. Moreover, future studies should evaluate the EMBRACE intervention through a process evaluation, exploring contextual factors, implementation processes, and mechanisms of impact. Finally, it would be important to design an intervention for healthcare professionals to ALS-families as they experience decreased job satisfaction and are at risk of burn-out.

## Supplementary Information

**Additional file 1.**

## Data Availability

The datasets generated and analyzed during the current study are not publicly available because they contain information that could compromise research participant privacy/consent, but they are available from the corresponding author on reasonable request.
