# Supplementary material for: A cross-sectional evaluation of acceptability of an online palliative rehabilitation program for family caregivers of people with amyotrophic lateral sclerosis and cognitive and behavioral impairments
Source: BMC Health Serv Res. 2022 May 24;22:697. doi: 10.1186/s12913-022-07986-4 (PMC9128325; doi:10.1186/s12913-022-07986-4)
Supplement: Supplementary file 1 — Additional file 1. [file 12913_2022_7986_MOESM1_ESM.docx]

**Additional file 1.** Interview guide for caregivers of persons with amyotrophic lateral sclerosis and cognitive and/or behavioral impairments.

| **Key questions** | **Elaborative questions** |
| --- | --- |
| **Tell me about how it has been for you to participate in the EMBRACE intervention?** | What did you gain from participating?  Was there anything you experienced that supported you? If yes, what?  What prompted you participate?  What did you miss from participating? |
| **How were you able to adapt the things you learned into your everyday life?** | How was EMBRACE useful to you in your everyday life?  How did you use the things your learned in EMBRACE in coping with everyday challenges?  Which challenges did you experience when trying to adapt the learned skills into your everyday life?  Generally, what did you think of EMBRACE? |
| (Looking at picture of every element in the intervention)  **Which elements were the most meaningful for you?** | Please elaborate why? |
| **What did you think about the videos?** | How many videos did you watch?  What was your impression of the content of the videos?  How did you use the videos during the intervention?  How did you use the videos as preparation before the meetings?  How did the topics fit your needs?  What did you think of the number of videos?  What did you think of the lengths of the videos?  What did you think of the way the videos were distributed to you after each meeting as preparation for the next meeting? |
| **How was it to meet the other participants?** | What you think of the group meetings?  How did you use the other participants?  What influence did the group composition have on your openness?  What was great about the meetings?  What did you think about the content in the meetings?  What did you learn?  What was not great about the meetings?  How did the virtual meetings work out?  What did you think about the size of the group?  What would you have liked differently about the meetings?  What did you think about the facilitator?  How did the length of the meetings fit you?  How did the time of the day fit you?  How did the frequency fit you?  How did the number of meetings fit you? |
| **What is your opination of the blended learning format, combining videos and virtual meetings?** | What did you think of the online format?  What did it mean to you that EMBRACE was online?  Which challenges did you experience with the online format?  How did you experience the coherence between the videos and the meetings?  How did you prioritize your time between watching the videos and participating in the meetings? |
| **How did you use the diary?** | What prompted you to use the diary/or not?  How did your dairy reflections support you through the intervention?  Prospectively, how would you use the diary?  How would you like to share the content of your diary with family and friends? |
| **How did you use the mindfulness exercises?** | What made you use the exercises/or not?  What impact did the exercises have on you?  In which situations did you use mindfulness? |
| **What did your opination regarding use of the ecomaps?** | What prompted you to draw the ECOMAPs/ or not?  How did the ECOMAPs effect you?  What was great about making ECOMAPS?  What was not that great about making ECOMAPs?  What did you think about making three ECOMAPs? |
| **What did you think about the opportunity to write messages to the other group members?** | Why did you use the chatroom/ or not?  What was great about the chatroom?  What was unfavorable about the chatroom? |
| **If you could change anything in EMBRACE, what would it be?** | What did you like most?  What did you not like?  What was the easiest thing?  What was the hardest thing?  All things considered, how has it been for you to participate? |
